# Rosmarinic Acid Induces Proliferation Suppression of Hepatoma Cells Associated with NF-κB Signaling Pathway

**DOI:** 10.31557/APJCP.2021.22.5.1623

**Published:** 2021-05

**Authors:** Yanjun An, Jiandong Zhao, Yourui Zhang, Wen Wu, Jiangtao Hu, Haosen Hao, Yu Qiao, Ying Tao, Liping An

**Affiliations:** *Department of Endoscopy Center, Institute of Shanxi Traditional Chinese Medicine, Hospital of Shanxi Traditional Chinese Medicine, Taiyuan, Shanxi, China. *

**Keywords:** Hepatocellular carcinoma (HCC), proliferation, HepG2 cells, Rosmarinic acid (RA)

## Abstract

**Background:**

Rosmarinic acid (RA) is a natural phenolic compound that acts as a Fyn inhibitor by 53 homology modeling of the human Fyn structure. Therefore, the apoptosis mechanism related to NF-κB signaling pathway induced by RA in HepG2 was investigated.

**Methods:**

The cell growth, apoptosis, and proliferation of HepG2 regulated by various concentrations of RA were studied. The proteins expression of MMP-2, MMP-9, PI3K, AKT, NF-κB, and apoptosis-related proteins Bax, Bcl-2, cleaved caspase-3 were detected.

**Results:**

RA significantly reduced proliferation rates, inhibited migration and invasion, and decreased the expressions of invasion-related factors, such as matrix metalloproteinase (MMP)-2 and MMP-9. TUNEL staining revealed that RA resulted in a dose-dependent increase of HepG2 cell apoptosis. In line with this finding, the expression of apoptosis suppressor protein Bcl-2 was downregulated and that of the pro-apoptotic proteins Bax and cleaved caspase-3 was increased. In addition, we found that the phosphatidylinositol 3-kinase (PI3K)/Akt/nuclear factor kappa B (NF-κB) signaling pathway was involved in RA-mediated inhibition of HepG2 cell metastasis.

**Conclusion:**

Our study identified that RA as a drug candidate for the treatment of HCC.

## Introduction

HCC (hepatocellular carcinoma) is the common tumor in the world, and its mortality rate is third among all cancers, just subsequent to gastric cancer and esophageal cancer (Bosetti et al., 2014; Camargo et al., 2014). Due to its difficult early diagnosis, easy early metastasis, rapid development and lack of effective therapy, the five-year survival of HCC was about 5~15% (Bosetti et al., 2014; El Serag HB and Rudolph, 2007). The pathogeny of HCC may be related to persistent liver damage, such as chronic HBV or HCV infection, exposure to heavy alcohol consumption, smoking or aflatoxin, or may be direct genotoxic substance, all of which are of significant relevance (El-Serag, 2012). Most patients sequentially develop hepatitis, fibrosis, cirrhosis, and then HCC. The biggest risk factor related to the progression of cancer formation in HCC is cirrhosis, which may come from chronic hepatic inflammation and liver injury for a long time about 20-40 years (El Serag and Rudolph, 2007). More than 80% HCC developed from fibrosis or cirrhotic livers, it also indicate long-term biological, chemical or physical damage that is playing an important role in hepatocytes carcinogenesis (Affo et al., 2017).

Rosmarinic acid (RA) is a natural phenolic compound that acts as a Fyn inhibitor by homology modeling of the human Fyn structure (Jelić et al., 2007). RA can be found in species of the Boraginaceae and Lamiaceae families, mainly in the leaves of Rosmarinus officinalis, from which it can be easily isolated; it can also be found in peppermint, lemon balm, oregano, sage, and thyme (Şengelen and Önay-Uçar, 2018; Juskowiak et al., 2018). The molecular structure of RA (chemical formula: C18H16O8) contains two benzene rings located at the molecule’s extremities and a pair of ortho-hydroxyl groups in each benzene ring. It has been reported that RA exerts a variety of beneficial biological properties, mainly antioxidant (Yang et al., 2013), anti-inflammatory (Chu et al., 2012), pro-apoptotic (Lin et al., 2007), and neuroprotective Kelsey et al., (2010) effects. Furthermore, recent studies have revealed that RA has antineoplastic activity in leukemia, hepatocellular carcinoma, gastric carcinoma, colorectal cancer, breast cancer, and small-cell carcinoma of the lung (Wu et al., 2013; Yesil-Celiktas et al., 2010). However, the effects of RA on tumor biological characteristics, such as proliferation, migration, and invasion of human hepatoma cells and their mechanisms, have not been clearly reported. 

The phosphatidylinositol 3-kinase (PI3K)/Akt/nuclear factor kappa B (NF-κB) signaling pathway is an important pathway in the regulation of tumorigenesis, and is significantly activated in HCC (Li et al., 2020). The activation of PI3K/Akt/NF-κB signaling cascades is inhibited by Fyn knockdown in primary astrocytes (Ko et al., 2015). Therefore, we hypothesized that RA may have an anti-HCC effect by inhibiting the PI3K/Akt/NF-κB signaling pathway.

Thus, the bio-functions induced by RA expression in HepG2 cells were studied The cell strain of human hepatoma HepG2 cell line, which is widely used to understand the HCC. The aim of the present study was to investigate the effect of RA on HepG2, and the roles of RA in NF-κB signaling pathway associated with the progression of HCC.

## Materials and Methods

*Cell line of HepG2*

The human hepatoma cell line HepG2 was obtained from ATCC (American Type Culture Collection), and cultured with DMEM medium (Gibco, Carlsbad, CA, USA) and 10% FBS (Gibco, Carlsbad, CA, USA) in a humidified incubator with 95% air, 5% CO_2_- humidified atmosphere at 37^o^C. In experiment group, cells were treated with different concentration of RA. RA was purchased from Aladdin (R109804; China).

*Western Blot Analysis*

The total protein from cells was extracted using RIPA lysis buffer, then concentrations of total proteins were quantified with bicinchoninic acid assay. Total protein (50 µg) was added into 12% SDS-PAGE gel, further transferred onto polyvinylidene difluoride membranes. 5% non-fat dry milk in Tween-20 (0.1%)-containing TBS was used to block the membranes, then which was incubated with primary antibodies, further incubated using the HRP (horseradish peroxidase)-conjugated second antibodies (1:1000; Cell Signaling Technology, Beverly, MA, USA). Blots were visualized by electrochemiluminescence detection system (Thermo Fisher Scientific, Waltham, MA, USA). 

The following primary antibodies were used: Fyn (ab125016; Abcam, USA), PI3K (4249S; Cell Signaling Technology, USA), MMP-2 (87809S; Cell Signaling Technology), MMP-9 (13667S; Cell Signaling Technology), Bax (3498S; Cell Signaling Technology), Bcl-2 (2772S; Cell Signaling Technology), cleaved caspase-3 (9661S; Cell Signaling Technology), caspase-3 (9662S; Cell Signaling Technology), phosphorylated Akt (p-Akt) (Sc-514032; Santa Cruz, USA), Akt (Sc-81434; Santa Cruz), NF-κB p65 (Sc-8008; Santa Cruz), β-actin (Sc-47778; Santa Cruz), and glyceraldehyde-3-phosphate dehydrogenase (GAPDH) (Sc-47724; Santa Cruz).

*Analyze viability of HepG2 cells*

The viability of HepG2 cells was detected using CCK-8 method. Briefly, cultured primary cortical neurons were cultured in plates (5×10^3^ cells/well). After 24 h of incubation, CCK-8 solutions were added into each well. After incubation for another 16 h, and then absorbance (450 nm) was measured with Microplate Reader. 

*Determine proliferation of HepG2 cells*

The proliferation of HepG2 cells were detected with XTT (sodium 3’-[1-(phenylaminocarbonyl)-3, 4-tetrazolium]-bis (4-methoxy-6-nitro) benzene sulfonic acid hydrate) (Roche Applied Science, Mannheim, Germany). After cultured in 96-well plates (3×103 cells/well) for 48 h, HepG2 cells were dealt with plasmids or siRNA, then cell proliferation assay was performed, the absorbance (450 nm) was measured with microtiter plate reader (Bio-Rad).

*BrdU assay and determine of mitotic entry*

The transfection of siRNA or plasmids was conducted during the interval of two blocks with thyidine for avoid the its potential effect on cell cycle. The synthesis of DNA was determined with BrdU labeling, BrdU-positive HepG2 cells were manually scored with immunofluorescence microscope. Furthermore, according to DNA staining using time-lapse videomicroscopy, the events of mitotic entry were recorded through observing condensation of DNA and morphology of nucleus. 

*Determimation of apoptosis*

Apoptotic rates of cells were determined with FITC Annexin-V Apoptosis Detection Kit I (BD, San Diego, California, USA). Briefly, cells were collected, washed with PBS and resuspended in binding buffer, double-stained with propidium iodide (PI) and Annexin V-FITC, followed by the apoptosis analysis using flow cytometry (BD Biosciences, San Jose, CA, USA). 

*Wound Healing Assay*

Cell migration was evaluated by using the wound healing assay as described previously. Briefly, HepG2 cells were seeded in 24-well plates at 5×10^4^ cells/well and allowed to adhere. Then, a wound was scratched by using a 200-μl pipette tip. After washing off the separated cells with phosphate-buffered solution (PBS), serum-free medium containing different concentrations of RA (0, 100, 200, and 400 μM) was added to the wells. The wound was observed at regular intervals between 0 and 48 hrs. Randomly selected areas were photographed by using a phase-contrast microscope (Olympus CKX41, Japan) and the wound area was calculated by ImageJ software (Bio-Rad, USA).

*Invasion Assay*

The invasion assay was conducted by using Corning^® ^BioCoat™ Matrigel^®^ Invasion Chambers (Catalog No. 354480; Corning, USA) with 8-μm pore chambers inserted into 24-well plates. HepG2 cells (5 × 10^4^ cells) were cultured in 500 μl of serum-free DMEM with RA in the inserts and 500 μl of DMEM containing 15% FBS in the bottom of wells. After 24 hrs of incubation, the inserts were washed three times with PBS and the cells were fixed with 4% paraformaldehyde for 10 min. After washing with PBS, the cells were stained with 0.1% crystal violet for 15 min. After another wash with PBS, the inner sides of the chamber were wiped with a cotton swab and images of the cells that invaded through the Matrigel^®^ were taken using a phase-contrast microscope. Finally, the number of invading cells was counted. 

*TUNEL and DAPI Staining*

HepG2 cells were seeded on a cover slide in a 24-well plate at a density of 3×10^4^ cells per well and kept in a CO_2_ incubator for overnight growth. Cells were treated with various concentrations of RA (0, 100, 200, and 400 μM) or vehicle for 24 hrs. Terminal deoxynucleotidyl transferase (TdT) dUTP nick-end labeling (TUNEL) staining was performed according to the manufacturer’s instructions (C1086; Beyotime Biotechnology, China). 

Briefly, the cells were fixed with 4% paraformaldehyde for 30 min, incubated for 5 min with 0.3% Triton X-100 in PBS, and then incubated with a reaction mixture containing terminal deoxynucleotidyl transferase and fluorescent labeling solution for 60 min according to the manufacturer’ s protocol. The cells were then stained with 4’ ,6-diamidino-2- phenylindole (DAPI) for 5 min and mounted using ProLongTM Gold antifade reagent (P36930; Thermo Fisher Scientific, USA). Stained cells were analyzed using a confocal microscope (LSM 800; Zeiss, Germany).

*Statistics*

The continuous variables with normally distribute were indicated with mean ± SD (standard deviation). GraphPad Prism 5.0 was used to conduct statistical analysis. For comparisons between two groups, Student’s t-test was performed. Moreover, one-way analysis of variance (ANOVA) with Kruskal-Wallis post hoc tests were performed for multiple group comparisons to compare and analyze quantitative data. Besides, abnormally distributed data between two groups were analyzed with Kruskal-Wallis analysis of variance method. P<0.05 was considered as difference with statistically significance.

## Results

*RA inhibits expression of Fyn*

**Figure 1:**
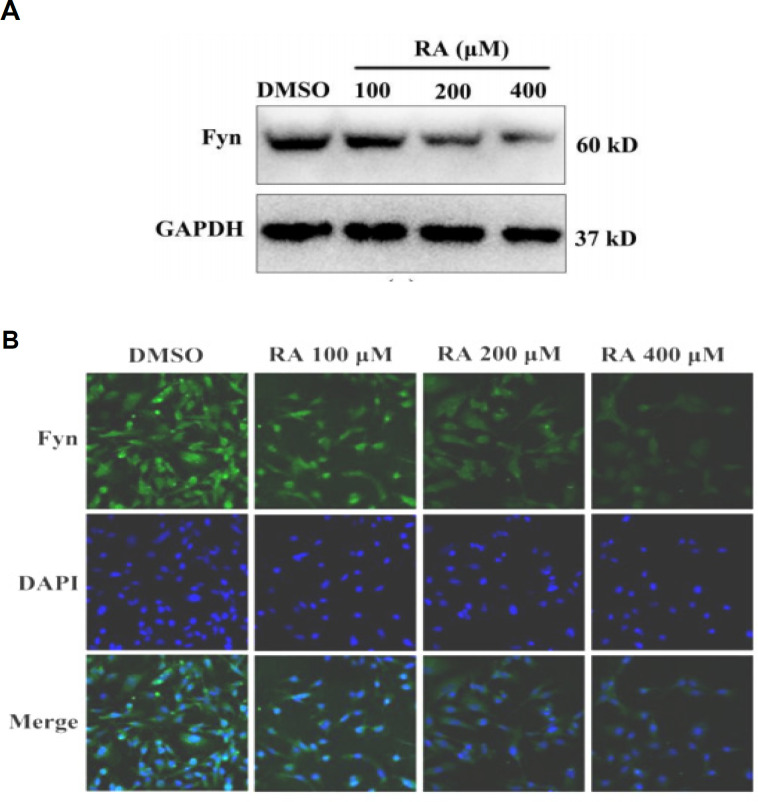
RA Inhibits Expression of Fyn. We firstly examined the effect of RA on the expression of Fyn in HepG2 cells. RA treatment reduced Fyn expression in HepG2 cells in a dose-dependent manner (A). Immunofluorescence staining confirmed that RA inhibits Fyn expression in HepG2 cells (B)

We firstly examined the effect of RA on the expression of Fyn in HepG2 cells. RA treatment reduced Fyn expression in HepG2 cells in a dose-dependent manner (Figure 1A). Immunofluorescence staining confirmed that RA inhibits Fyn expression in HepG2 cells (Figure 1B).

*RA inhibits hepatoma cell proliferation*

**Figure 2:**
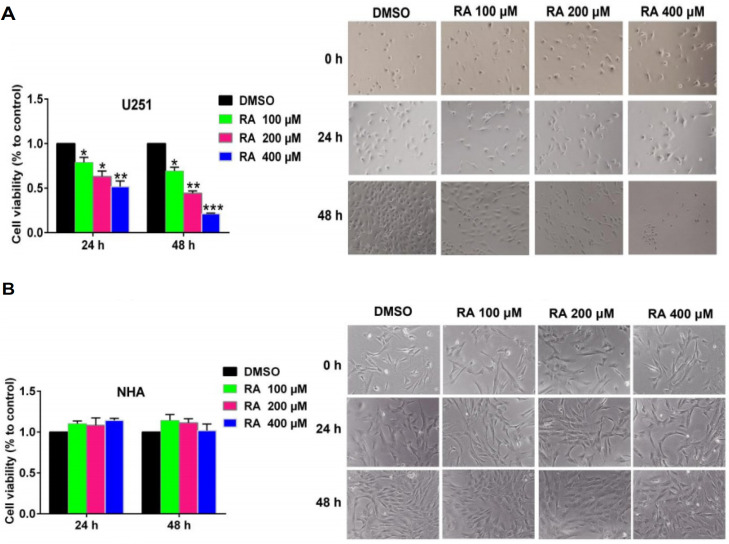
RA Inhibits Hepatoma Cell Proliferation. Next, we examined the effect of RA on hepatoma cell proliferation using the CCK-8 assay. RA treatment inhibited cell proliferation in a time- and dose-dependent manner in HepG2 cells. RA-induced cytotoxicity was also observed under a microscope. The images show that as the concentration of RA increased, the density of hepatoma cells decreased. Moreover, the cells shrank and died more. These observations were in line with the results of the CCK-8 assay (A). As a control, we also examined the effect of RA on NHA. RA treatment for 24 hrs or 48 hrs did not significantly affect NHA cell viability and morphology (B). These results indicated that RA specifically inhibited the proliferation of hepatoma cells, with no significant effect on the proliferation of NHA

Next, we examined the effect of RA on hepatoma cell proliferation using the CCK-8 assay (Figure 2). RA treatment inhibited cell proliferation in a time- and dose-dependent manner in HepG2 cells. RA-induced cytotoxicity was also observed under a microscope. The images show that as the concentration of RA increased, the density of hepatoma cells decreased. Moreover, the cells shrank and died more. These observations were in line with the results of the CCK-8 assay (Figure 2A). As a control, we also examined the effect of RA on NHA. RA treatment for 24 hrs or 48 hrs did not significantly affect NHA cell viability and morphology (Figure 2B). These results indicated that RA specifically inhibited the proliferation of hepatoma cells, with no significant effect on the proliferation of NHA.

*RA inhibits hepatoma cell migration*

**Figure 3:**
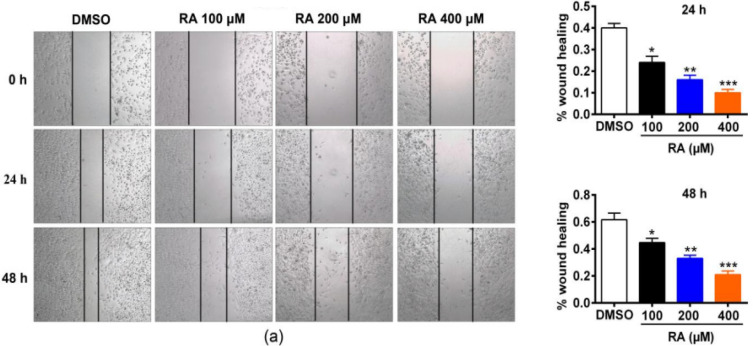
RA Inhibits Hepatoma Cell Migration. Metastasis of rapidly migrating tumor cells is the main cause of death for most patients with cancer [19]. Therefore, inhibiting migration could be an important strategy to prevent tumor metastasis. We examined the effect of RA on hepatoma cell migration. HepG2 cells showed reduced migration after treatment with RA for 24 hrs or 48 hrs at different concentrations. Specifically, in the control group of the HepG2 cell line, wound healing reached 40% after 24 hrs, while it only reached 24%, 18%, and 10% after treatment with 100, 200, and 400 µM RA, respectively. At 48 hrs, wound healing of control HepG2 cells reached 60%, but 42%, 30%, and 20% after treatment with 100, 200, and 400 µM RA, respectively

Metastasis of rapidly migrating tumor cells is the main cause of death for most patients with cancer (Yachida et al., 2010). Therefore, inhibiting migration could be an important strategy to prevent tumor metastasis. We examined the effect of RA on hepatoma cell migration (Figure 3). HepG2 cells showed reduced migration after treatment with RA for 24 hrs or 48 hrs at different concentrations (Figure 3). Specifically, in the control group of the HepG2 cell line, wound healing reached 40% after 24 hrs, while it only reached 24%, 18%, and 10% after treatment with 100, 200, and 400 µM RA, respectively. At 48 hrs, wound healing of control HepG2 cells reached 60%, but 42%, 30%, and 20% after treatment with 100, 200, and 400 µM RA, respectively.

*RA inhibits hepatoma cell invasion*

**Figure 4:**
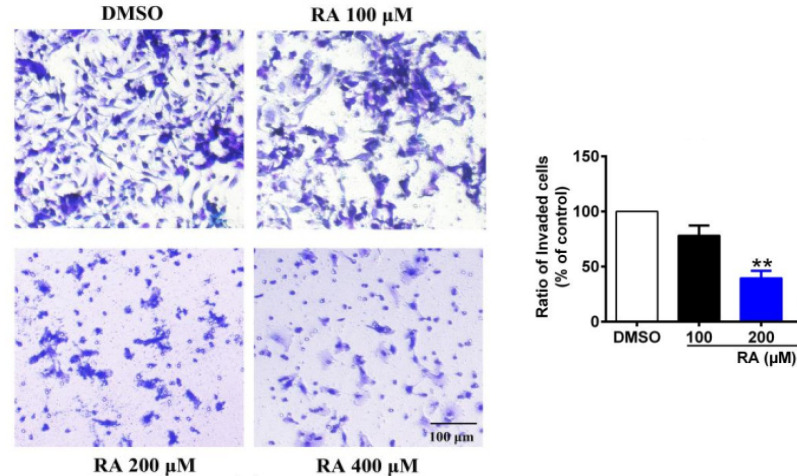
RA Inhibits Hepatoma Cell Invasion. The high invasion capability of hepatoma cells is the main reason for the high refractory and recurrence rates of HCC. Therefore, we tested the effect of RA on hepatoma cell invasion using a Matrigel^®^ invasion assay. RA significantly inhibited invasion through the Matrigel^®^. Compared with the control group, the percentage of invading HepG2 cells was decreased by 21.7%, 60.3%, and 76.4% after treatment with 100, 200, and 400 µM RA for 24 hrs, respectively

The high invasion capability of hepatoma cells is the main reason for the high refractory and recurrence rates of HCC. Therefore, we tested the effect of RA on hepatoma cell invasion using a Matrigel^®^ invasion assay (Figure 4). RA significantly inhibited invasion through the Matrigel^®^. Compared with the control group, the percentage of invading HepG2 cells was decreased by 21.7%, 60.3%, and 76.4% after treatment with 100, 200, and 400 µM RA for 24 hrs, respectively (Figure 4).

**Figure 5:**
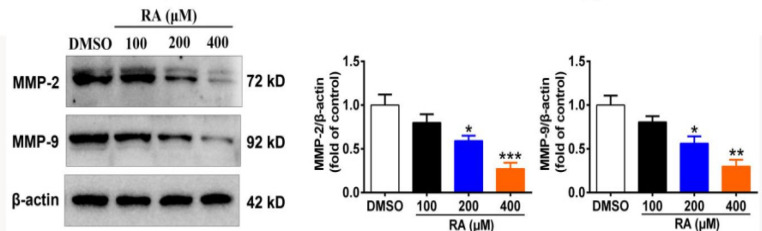
RA Inhibits Hepatoma Cell Invasion. Moreover, we detected the expression of MMP-2 and MMP-9 by western blot. RA dose-dependently inhibited the expression of MMP-2 and MMP-9 in HepG2 cells. Compared with the control group, the expression of MMP-2 in HepG2 cells was decreased by 20.0%, 40.7%, and 62.7%, and that of MMP-9 was decreased by 19.9%, 43.7%, and 70%, after treatment with 100, 200, and 400 µM for 24 h, respectively. These results suggested that RA inhibited hepatoma cell invasion and migration by reducing the expression of MMPs, thereby providing pathways for the invasion and metastasis of hepatoma cells

Previous studies have reported the role of matrix metalloproteinase-2 (MMP-2) and MMP-9 in cancer development, including tumor cell growth, migration, invasion, and metastasis, and particularly so in HCC (Xu et al., 2018; Zheng et al., 2019). We detected the expression of MMP-2 and MMP-9 by western blot. RA dose-dependently inhibited the expression of MMP-2 and MMP-9 in HepG2 cells (Figure 5). Compared with the control group, the expression of MMP-2 in HepG2 cells was decreased by 20.0%, 40.7%, and 62.7%, and that of MMP-9 was decreased by 19.9%, 43.7%, and 70% (Figure 5), after treatment with 100, 200, and 400 µM for 24 h, respectively. These results suggested that RA inhibited hepatoma cell invasion and migration by reducing the expression of MMPs, thereby providing pathways for the invasion and metastasis of hepatoma cells.

*RA increases hepatoma cell apoptosis*

**Figure 6:**
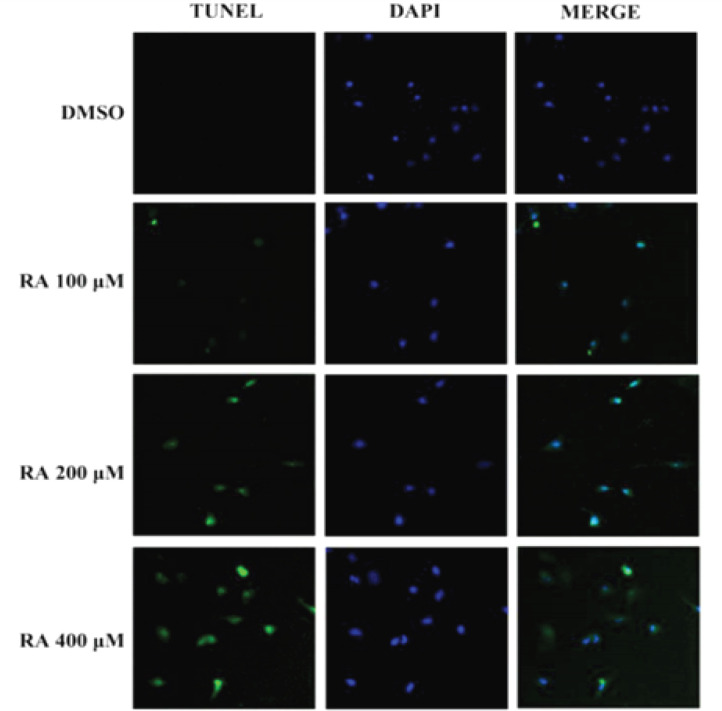
RA Increases Hepatoma Cell Apoptosis. We investigated whether RA could induce apoptosis. TUNEL assay revealed that RA induced apoptosis significantly in a dose-dependent manner in HepG2 cells

**Figure 7:**
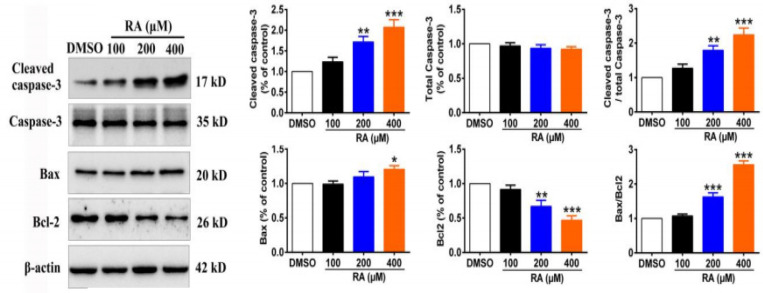
RA Increases Hepatoma Cell Apoptosis. We found that RA treatment increased the expression of cleaved caspase-3 and Bax and reduced the expression of Bcl-2 in HepG2 cells. In addition, RA treatment increased the relative ratio of Bax/Bcl-2 as well as cleaved caspase3/caspase3, indicating RA-induced apoptosis in hepatoma cells. Compared with the control group, the relative ratio of Bax/Bcl-2 in HepG2 cells increased 7.6%, 63.0%, and 156.3%, after treatment with 100, 200, and 400 µM RA for 24 hrs, respectively. In addition, the relative ratio of cleaved caspase3/caspase3 in HepG2 cells increased 27.3%, 79.6%, and 124.3%, after treatment with 100, 200, and 400 µM RA for 24 hrs

Apoptosis plays a crucial role in cancer treatment (Torres et al., 2011). Therefore, we investigated whether RA could induce apoptosis. TUNEL assay revealed that RA induced apoptosis significantly in a dose-dependent manner in HepG2 cells (Figure 6). The apoptosis suppressor protein Bcl-2 and the pro-apoptotic proteins Bax, cleaved caspase-3, and caspase-3 are key in the process of apoptosis in hepatoma cells. We found that RA treatment increased the expression of cleaved caspase-3 and Bax and reduced the expression of Bcl-2 in HepG2 cells (Figure 7). In addition, RA treatment increased the relative ratio of Bax/Bcl-2 as well as cleaved caspase3/caspase3, indicating RA-induced apoptosis in hepatoma cells. Compared with the control group, the relative ratio of Bax/Bcl-2 in HepG2 cells increased 7.6%, 63.0%, and 156.3%, after treatment with 100, 200, and 400 µM RA for 24 hrs, respectively. In addition, the relative ratio of cleaved caspase3/caspase3 in HepG2 cells increased 27.3%, 79.6%, and 124.3%, after treatment with 100, 200, and 400 µM RA for 24 hrs (Figure 7).

*RA inhibits the PI3K/Akt/NF-κB signaling pathway in hepatoma cells*

**Figure 8:**
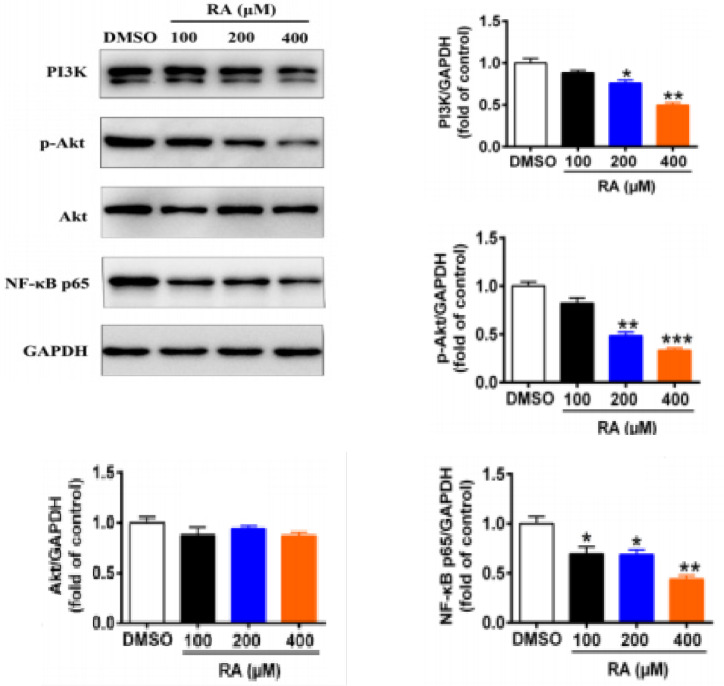
RA Inhibits the PI3K/Akt/NF-κB Signaling Pathway in Hepatoma Cells. We examined the effect of RA on the PI3K/Akt/NF-κB signaling pathway. We found that treatment with 100, 200, and 400 µM RA for 24 hrs dramatically decreased the protein expression of PI3K, p-Akt, and NF-κB in HepG2 cells compared to control cells. Specifically, the expression of PI3K was decreased by 11.7%, 24%, and 50.3% in HepG2 cells, respectively. In HepG2 cells, the expression of p-Akt was decreased by 18.0%, 51.7%, and 66.7% and that of NF-κB p65 by 30.7%, 31.4%, and 56%, respectively. Collectively, these results suggested that RA might inhibit proliferation, migration, and invasion and induce apoptosis of hepatoma cells via the PI3K/Akt/NF-κB signaling pathway

PI3K/Akt/NF-κB signaling plays a vital role in cell proliferation, survival, and metabolism, and is constitutively activated in most tumors, including hepatoma (Tang et al., 2016; Qiu et al., 2016). Therefore, we examined the effect of RA on the PI3K/Akt/NF-κB signaling pathway. We found that treatment with 100, 200, and 400 µM RA for 24 hrs dramatically decreased the protein expression of PI3K, p-Akt, and NF-κB in HepG2 cells compared to control cells (Figure 8). Specifically, the expression of PI3K was decreased by 11.7%, 24%, and 50.3% in HepG2 cells, respectively. In HepG2 cells, the expression of p-Akt was decreased by 18.0%, 51.7%, and 66.7% and that of NF-κB p65 by 30.7%, 31.4%, and 56%, respectively. Collectively, these results suggested that RA might inhibit proliferation, migration, and invasion and induce apoptosis of hepatoma cells via the PI3K/Akt/NF-κB signaling pathway.

## Discussion

Here, we explored the effects of RA on the biological characteristics of the hepatoma cell line HepG2. We found that RA inhibited proliferation, migration, and invasion and induced apoptosis in HepG2 cells. In general, the effects were dose-dependent. We also found that RA significantly reduced the protein levels of MMP-2 and MMP-9, which promote migration and induce invasion, and of the apoptosis suppressor protein Bcl-2; and increased the levels of pro-apoptotic proteins, such as cleaved caspase-3 and Bax in HepG2 cells. Previous study has reported that the PI3K/Akt/NF-κB signaling pathway plays an important role in the growth, proliferation, migration, and invasion of HCC (Cheng et al., 2006). Our current study further recognizes the importance of PI3K/Akt/NF-κB signaling in HCC.

In recent years, the use of traditional Chinese medicine to treat tumors has attracted increasingly attention of researchers owing to the significant curative efficacy and relatively fewer side effects. RA is a medicinal herb that not only is easy to isolate, but can also be ingested from food or tea (Petersen et al., 2003). RA has been reported to have antioxidant, antiviral, and anti-inflammatory effects and has been used in an Egyptian herbal tea for the prevention of cancer (Elansary and Mahmoud, 2015). Furthermore, RA significantly improves the efficiency of radiotherapy by exerting a radiosensitizing effect on tumor cells (Alcaraz et al., 2014). In addition, it enhances chemosensitivity of resistant gastric carcinoma cells to 5-Fu by downregulating miR-642a-3p and miR-6785-5p and increasing FOXO4 expression (Yu et al., 2019). Various studies have shown that RA has antineoplastic activity in leukemia, hepatocellular carcinoma, gastric carcinoma, colorectal cancer, breast cancer, and small-cell carcinoma of the lung. However, the effects of RA on the biological characteristics of HCC is limited. Our study showed that RA inhibited the expression of Fyn in a dose-dependent manner in HepG2 cells. This provides a theoretical basis for its anti-tumor effects in HCC. High invasion and rapid proliferation are the main reasons for refractory HCC and poor prognosis of HCC. Cell viability assay and morphological observation revealed that RA inhibited the growth of HCC in a time- and dose-dependent manner. At the same time, it little effect on NHA cell proliferation and morphology. This suggested that RA may be a promising anti-tumor drug based on its weak toxicity. Our study also demonstrated that RA inhibited HCC cell invasion. Since MMP-2 and MMP-9 are important regulators of invasion, we also examined the effect of RA on their expression levels. Western blot showed that treatment with RA decreased MMP-2 and MMP-9 expression in HCC cells. The main feature of tumor metastasis is the migration of cancer cells from the initial tumor site to the circulatory system or lymphatic system (McCall et al., 2018). Therefore, inhibiting tumor cell migration could reduce metastasis. In our study, RA inhibited migration of HepG2 cells. Considering that apoptosis is a crucial anti-tumor mechanism (Shin et al., 2020), we investigate whether RA could induce apoptosis in HCC cells. The percentage of apoptotic HepG2 cells was significantly increased after treatment with RA. As Bcl-family proteins are main regulators of apoptosis, we also examined the effect of RA on the expression of Bax and Bcl-2. RA increased Bax expression and decreased Bcl-2 expression in HCC cells. Correspondingly, the expression of cleaved (activated) caspase-3, but not that of total caspase-3, was increased by RA. In addition, the Bax/Bcl-2 and cleaved caspase-3/caspase-3 ratios were increased by RA, thus providing a mechanistic basis for the induction of apoptosis by RA in HCC cells. These results are similar to the reported role of RA in colorectal cancer (Han et al., 2018). Numerous studies have shown that the PI3K/Akt/NF-κB pathway is closely related to HCC progression (Fahey et al., 2019; Nakabayashi and Shimizu, 2012). In line with these reports, our results showed that the PI3K/Akt/NF-κB signal pathway is involved in the anti-tumor effects of RA in HCC.

In conclusion, taken together, these findings suggest that the anti-tumor effects of RA in HCC might be mediated by Fyn inhibition. The detailed mechanisms warrant further investigation in vitro and in vivo.

## Author Contribution Statement

YJA, and JDZ designed this research work. YRZ interpreted data. YJA, WW, JTH, HH, and YQ performed experiments. YT provided technical assistance. YJA, and LPA analyzed raw data. YJA substantively revised the manuscript, and wrote original draft. All authors read and approved the final manuscript.

## Scientific statement

It was not approved by any scientific Body or is part of an approved student thesis.
